# The impact of child maltreatment on non-suicidal self-injury: data from a representative sample of the general population

**DOI:** 10.1186/s12888-018-1754-3

**Published:** 2018-06-08

**Authors:** Rebecca C. Brown, Stefanie Heines, Andreas Witt, Elmar Braehler, Joerg M. Fegert, Daniela Harsch, Paul L. Plener

**Affiliations:** 1 Department of Child and Adolescent Psychiatry/Psychotherapy, University of Ulm, Steinhoevelstr, 5, 89075 Ulm, Germany; 2 Department of Psychosomatic Medicine and Psychotherapy, University Medical Center of the Johannes Gutenberg University of Mainz, Langenbeckstraße 1, 55131 Mainz, Germany; 3 Department of Medical Psychology and Medical Sociology, University of Leipzig, Leipzig, Germany; 4 Department of Child and Adolescent Psychiatry, Medical University of Vienna, Waehringerguertel 18-20, 1090 Vienna, Austria

**Keywords:** Child maltreatment, Non-suicidal self-injury, NSSI, Child abuse and neglect

## Abstract

**Background:**

Child maltreatment is an identified risk factor for Non-Suicidal Self-Injury (NSSI). The aim of the current study was to investigate effects of different types of maltreatment, and mediating effects of depression and anxiety on NSSI in the general population.

**Methods:**

A representative sample of the German population, comprising *N* = 2498 participants (mean age = 48.4 years (SD = 18.2), 53.3% female) participated in this study. Child maltreatment was assessed using the Childhood Trauma Questionnaire (CTQ),NSSI was assessed with a question on lifetime engagement in NSSI, depressive symptoms were assessed by the Patient Health Questionnaire (PHQ-2) and anxiety symptoms by the General Anxiety Disorder questionnaire (GAD-2).

**Results:**

Lifetime prevalence of NSSI in this sample was 3.3, and 30.8% reported at least one type of child maltreatment. Participants in the NSSI group reported significantly more experiences of child maltreatment. Emotional abuse was endorsed by 72% of all participants with NSSI. A path analytic model demonstrated an unmediated direct effect of emotional neglect, a partially mediated effect of emotional abuse, and a fully mediated effect of sexual abuse and physical neglect by depression and anxiety on NSSI.

**Conclusions:**

Especially emotional neglect and abuse seem to play a role in the etiology of NSSI above and beyond depression and anxiety, while sexual and physical abuse seem to have a rather indirect effect.

## Background

Child maltreatment (emotional, physical, and sexual abuse, as well as physical and emotional neglect) has shown to be associated with a large variety of different mental- and physical health problems throughout the lifespan [1, 2]. The experience of at least one type of child maltreatment was reported by around 30% of the general population of Germany in two independent studies [3, 4]. Different mental health problems like depressive disorders, suicide attempts, and drug abuse have been identified as long-term consequences of child maltreatment [1]. Furthermore, child maltreatment has been identified as risk-factor for Nonsuicidal Self-Injury (NSSI) and self-harm in several studies (for review see [5]). NSSI is defined as the deliberate damaging of the surface of the skin (e.g. cutting, scratching, or burning) without suicidal intent [6]. Studies investigating NSSI in the general population have found NSSI to be very common among adolescents, with international lifetime prevalence rates of around 18% [7]. However, studies from adult populations indicate that NSSI is less prevalent in mid- to late adulthood. A study from the general population in the US using random digit dialing reported a lifetime prevalence of 5.9 and 0.9% within the last year [8]. These results correspond well to a recent study of a representative sample of the German general population, finding a lifetime prevalence of 3 and 0.3% within the last year [9].

Several studies have investigated the relationship between child maltreatment and NSSI, however mainly in adolescent samples. So far, most consistent evidence has been found for emotional abuse and neglect to be related to the engagement in NSSI. In a systematic review of the literature, Lang and Sharma-Patel [5] found consistent evidence for emotional neglect to predict NSSI. A very recent study [10] reported a direct association of emotional abuse and NSSI, while the effect of sexual and physical abuse was mediated completely by emotion expressivity (i.e. difficulties in expressing emotions appropriately) and emotion coping (ability to cope with negative emotions).

These results are in line with mixed results on sexual and physical abuse in previous studies. In a meta-analysis including 45 studies on the association of child sexual abuse and self-injury, Klonsky and Moyer [11] found a modest relationship between child sexual abuse and NSSI. It has been shown in several other studies, that the association between NSSI and sexual abuse was (almost) completely mediated by factors like low self-esteem [12], dissociation [13], alexithymia [14], self-criticism [15], and posttraumatic stress symptoms [16]. A more recent systematic review [17] reported similar outcomes. Regarding physical abuse, results from studies are also rather mixed, with some results pointing towards a significant relationship [18, 19], while others did not find significant associations [10, 20]. One study [15] also found a significant effect of physical neglect on NSSI in a sample of adolescents.

These rather mixed results might be due to several mediating and moderating factors in the relationship between child abuse and neglect and engagement in NSSI. According to the Developmental Psychopathology Framework [21], especially negative experiences with primary caregivers can lead to deficits in emotion regulation and neurophysiological dysregulation, which can then in turn lead to engaging in maladaptive coping-strategies, like NSSI. However, this relationship seems to be of a rather complex nature, as different types of maltreatment may lead to impairment of different aspects of emotion regulation and might therefore have differential impact on NSSI [22].

While NSSI can occur without further psychopathology [23], it mostly co-occurs with other symptoms and disorders, such as depression and anxiety [24, 25]. Furthermore, a large body of research found depression and anxiety to be risk-factors for NSSI (for review see [26]), but these are also known to occur as a consequence of child maltreatment [1]. These factors might therefore also play a mediating role between the experience of child maltreatment and engaging in NSSI. A study by Glassman and colleagues, for example, found depression to be mediating the effect of child maltreatment on NSSI [15].

In summary, most studies testing the association of child maltreatment and NSSI were conducted in rather young clinical or convenience samples, while data from representative samples in adult populations are still scarce. Analyses of samples in the general population are important, as NSSI but also child maltreatment might be overrepresented in clinical and convenience samples. By recruiting a random, representative sample of the general population, evidence of results from previous studies including selective samples can be strengthened and new conclusions on the effects of child maltreatment on NSSI across the lifespan in a non-clinical population can be drawn. The aim of the current study was to investigate the association of different types of child maltreatment and NSSI in a sample of the general population of Germany. In detail, the differential impact of perceived severity and type of maltreatment, as well as a possible mediation of depressive and anxiety symptoms were of interest. It was hypothesized that (1) having experienced any type of child maltreatment would lead to a higher vulnerability for engaging in NSSI, (2) a cumulating effect of different types of child maltreatment on lifetime NSSI would be found, (3) especially emotional abuse and neglect would directly affect NSSI, and (4) that physical abuse and –neglect, as well as sexual abuse, would be mediated by depression and anxiety symptoms.

## Methods

Using a random route procedure, a representative sample of the German population was acquired by a demographic consulting company (USUMA, Berlin, Germany) between September and November 2016. Households of every third residence in a randomly chosen geographical area were invited to participate in the study. Households were approached by door to door in person recruitment. In multi-person households, participants were randomly selected using a Kish-Selection-Grid. After initial recruitment contact, research staff scheduled a time convenient for the participant to complete questionnaires. Inclusion criteria were a minimum age of 14 and sufficient knowledge of the German language. Of 4902 designated addresses, 2510 households participated in the study. Main reasons for non-participation were that participants were not present or refusal to participate. Responses were anonymous. In a first step, socio-demographic information was gathered in an interview-format by research staff. All other information was obtained via paper and pencil questionnaires, with research staff being available for questions.

The study was conducted in accordance with the Declaration of Helsinki, and fulfilled the ethical guidelines of the International Code of Marketing and Social Research Practice of the International Chamber of Commerce and of the European Society of Opinion and Marketing Research. All participants (and in case of minorstheir caregivers, 3.4% of the sample) gave informed written consent. The study was approved by the Ethics Committee of the Medical Department of the University of Leipzig.

### Measures

The prevalence of five types of child maltreatment was assessed using the 28 item brief version of the Childhood Trauma Questionnaire (CTQ) [27, 28]. The CTQ is a screening measure for the assessment of child maltreatment. It contains five subscales each assessed by five items, including sexual, (e.g. “someone tried to make me do sexual things or watch sexual things”) emotional, (e.g. “people in my family said hurtful or insulting things to me”) and physical abuse (e.g. “I was punished with a belt, a board, a cord, or some other hard object”),as well as emotional, (e.g. “I felt loved” (reversed)) and physical neglect (e.g. “I didn’t have enough to eat”). Additionally, three items assess whether participants tend to trivialize problematic experiences within their family (e.g. “There was nothing I would have wished to be different in my family”). Good psychometric properties of the German version of the CTQ were demonstrated by Klinitzke and colleagues [27], with internal consistencies ranging between Cronbach’s Alpha = .62 (physical neglect) and Cronbach’s Alpha = .96 (sexual abuse) for all subscales. The intra-class coefficient for an interval of six weeks was 0.77 for the overall scale and between 0.58 and 0.81 for subscales. In the current sample, internal consistencies were Cronbach’s Alpha = .55 for physical neglect, Cronbach’s Alpha = .82 for emotional neglect and emotional abuse, respectively, Cronbach’s Alpha = .83 for physical abuse, and Cronbach’s Alpha = .91 for sexual abuse. Based on norm data by Bernstein and colleagues [29], severity scores for each subscale can be calculated, ranging from “none -minimal”, “minimal-moderate”, “moderate-severe”, to “severe-extreme”. These categories were used in the current study, when calculating χ^2^ tests between participants with or without NSSI. Continuous scores of each scale of the CTQ were entered in the path analytic model.

Lifetime prevalence of NSSI was assessed by a question (“Have you ever intentionally harmed yourself, without the intention to die?”), which was taken from the Self-Injurious Thoughts and Behavior Interview (SITBI, [30]) in its validated German version (Fischer et al., 2014). The use of a paper and pencil version of the SITBI in its German version has been shown to provide reliable results in a former study [9].

Depressive symptoms were assessed with the German version of the screening tool Patient Health Questionnaire (PHQ-2), focusing on the depressive symptoms ‘low mood’ and ‘loss of interest’. Scores can reach values from 0 to 6, with a cut-off of values higher than 3 leading to values of sensitivity of 87% and specificity of 78% for major depression [31]. The internal consistency in the current sample was Cronbach’s Alpha = .81. Anxiety symptoms were screened for using the GAD-2 (General Anxiety Disorder questionnaire). Like the PHQ-2, the questionnaire consists of two items, with possible values from 0 to 6 and a cut-off of 3. This cut-off is sensitive for screening for generalized anxiety disorders (86%), panic disorders (76%), social anxiety disorder (70%) and moderately for PTSD (59%). It is specific for all four types of anxiety disorders mentioned above (81–83%), with an internal consistency of Cronbach’s alpha = .82 (Cronbach’s alpha = .79 in the current sample) [32].

### Participants

Of the *N* = 2510 participants, *n* = 12 participants were excluded due to missing data on NSSI. Participants were on average 48.4 years old (SD = 18.2) and 53.3% were female. The sample was representative for the German population with regard to age and gender. Of the entire sample, 21.7% reported to have completed high-school with ‘Abitur’, which qualifies for entering university, 5.3% reported to be currently unemployed, 54.5% lived with a partner, and 3.2% reported a place of birth outside of Germany. Average depression and anxiety scores of the entire sample were under the clinical cut-off of 3. A detailed description of participants with regard to child maltreatment and psychiatric symptoms by age group can be seen in Table 1.

**Table 1:** Experience of child maltreatment and psychiatric symptoms of participants divided by age groups

	14–19 years	20–29 years	30–39 years	40–49 years	50–59 years	60–69 years	70+ years
Child maltreatment							
Emotional abuse	5.7% (*N* = 8)	5.7% (*N* = 19)	6.9% (*N* = 27)	9.1% (*N* = 36)	7.8% (*N* = 38)	3.6% (*N* = 14)	5.7% (*N* = 20)
Physical abuse	7.1% (*N* = 10)	4.2% (*N* = 14)	6.1% (*N* = 24)	5.5% (*N* = 22)	8.2% (*N* = 40	6.2% (*N* = 24)	8.9% (*N* = 31)
Sexual abuse	5.0% (*N* = 7)	4.2% (*N* = 14)	6.9% (*N* = 27)	11.1% (*N* = 44)	9.2% (*N* = 45)	7.0% (*N* = 27)	6.3% (*N* = 22)
Emotional neglect	6.4% (*N* = 9)	12.7% (*N* = 42)	12.4% (*N* = 49)	13.1% (*N* = 52)	17.9% (M = 87)	10.9% (*n* = 42)	14.3% (*N* = 50)
Physical neglect	5.7% (*N* = 8)	16.9% (*N* = 56)	17.5% (*N* = 69)	18.7% (*N* = 74)	22.6% (*N* = 110)	20.9% (*N* = 81)	45.8% (*N* = 160)
Psychiatric symptoms							
Anxiety	6.4% (*N* = 9)	6.3% (*N* = 21)	7.6% (*N* = 30)	8.8% (*N* = 35)	10.7% (*N* = 52)	4.8% (*N* = 19)	6.1% (*N* = 21)
Depression	7.9% (*N* = 11)	6.4% (*N* = 21)	6.4% (*N* = 25)	8.3% (*N* = 33)	9.3% (*N* = 45)	5.1% (*N* = 20)	7.8% (*N* = 27)
NSSI	7.8% (*N* = 11)	7.5% (*N* = 25)	5.1% (*N* = 20)	3.5% (*N* = 14)	1.6% (*N* = 8)	0.8% (*N* = 3)	0.6% (*N* = 2)

Child maltreatment: at least moderate to severe level. Anxiety and depression levels: GAD-2 and PHQ-2 scores above clinical cut-off, NSSI: lifetime engagement NSSI

### Statistical analyses

Statistical analyses were conducted using SPSS version 21 and MPlus Version 7.31. Differences between the co-occurrence of child maltreatment and NSSI, and differences in the severity of child maltreatment were calculated using χ^2^ tests. Different types of child maltreatment were were weakly to moderately inter-correlated (*r* = 0.27 for emotional neglect and sexual abuse to *r* = 0.65 for emotional and physical neglect). For this reason, a path analytic model for the relationship of different types of child maltreatment as independent variables (continuous scores, and allowing for inter-correlation of those variables) with NSSI as the dependent variable and depression and anxiety scores (continuous scores) as mediating variables was calculated using MPlus. Model fit was calculated via the weighted root mean square residual (WRMR), which is best used for modelling categorical variables [33]. A WRMR< .90 implies good model fit for continuous and categorical variables [34]. As the NSSI and non-NSSI group differed significantly concerning age and gender, age and gender were included as covariates in the model. Standardized coefficients of the model are reported.

## Results

A total of 83 participants (3.3%) reported lifetime engagement in NSSI. Concerning child abuse, 30.8% reported having experienced at least one type of child maltreatment, with 16.8% reporting one type of maltreatment and 14.0% of the overall sample reported several types of maltreatment. The most common type of at least moderate to severe maltreatment was physical neglect (22.4%), followed by emotional neglect (13.2%), sexual abuse (7.6%), physical abuse (6.7%), and emotional abuse (6.5%). For further details on maltreatment data see Witt et al. [4].

### Differences between participant in the NSSI and the non-NSSI group

It was hypothesized that (1) having experienced any type of child maltreatment would lead to a higher vulnerability for engaging in NSSI, (2) a cumulating effect of different types of child maltreatment on lifetime NSSI would be found. Around two thirds (65.1%) of participants in the NSSI group reported having experienced at least one type of child maltreatment. This was significantly different from 29.7% of participants in the non-NSSI group (χ^2^ = 46.93, *p* < .001). Furthermore, 48.2% of participants in the NSSI group reported having experienced multiple types of child maltreatment, while this was true for 12.8% of the non-NSSI group (χ^2^ = 83.38, *p* < .001).

**Fig. 1:**
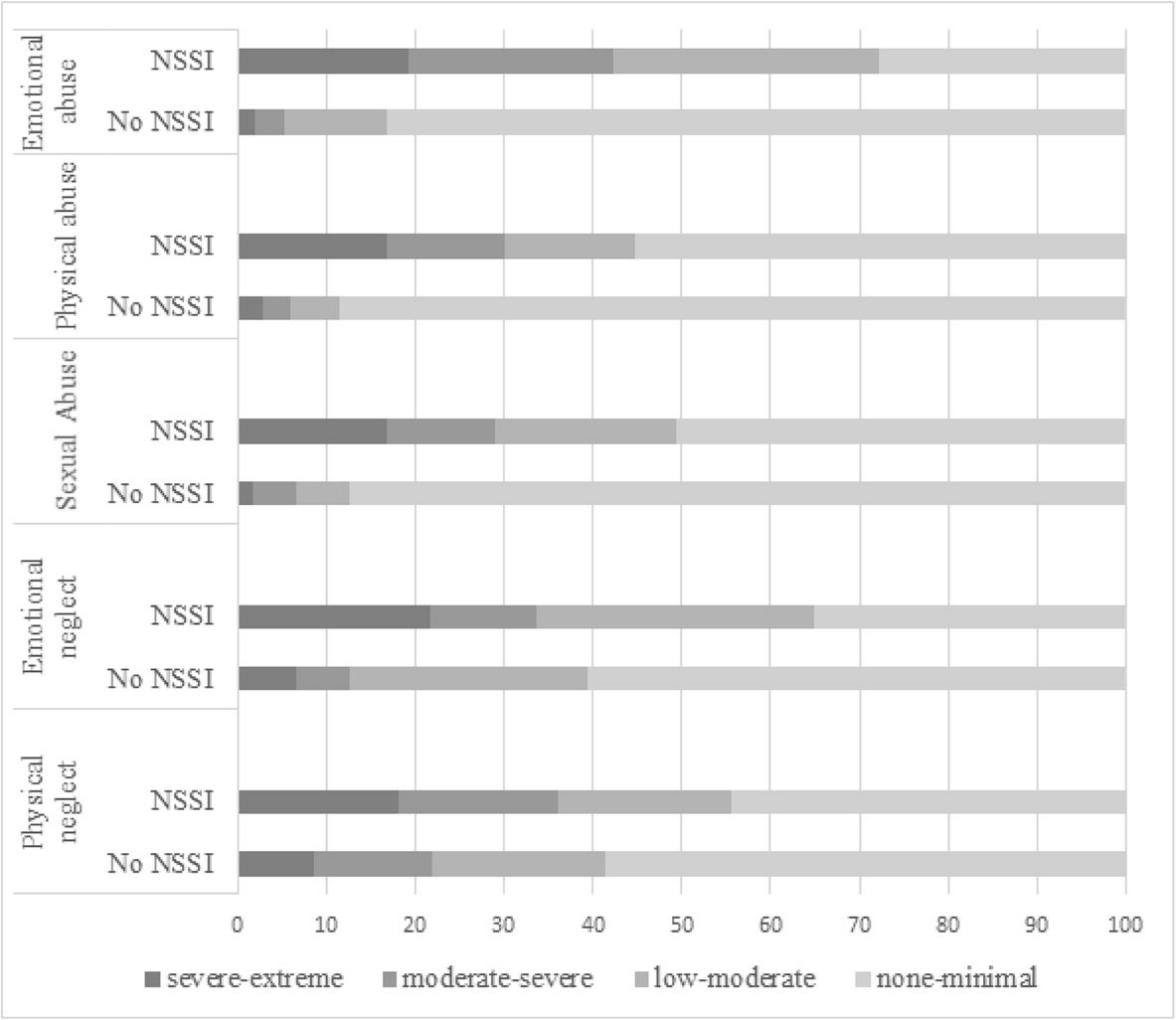
Different types of maltreatment by severity in association with lifetime engagement in NSSI. *Note*: displayed are percentages of participants meeting CTQ-scores in corresponding categories (severe-extreme to none-minimal)

With regards to different types of child maltreatment, participants with NSSI generally reported higher levels of child maltreatment in all areas (see Fig. 1). At least moderate to severe emotional abuse was reported by 42.2% of participants in the NSSI group vs. 5.3% in the non-NSSI group (χ^2^ = 223.5, *p* < .001). Physical abuse of at least moderate to severe level was reported by 32.2% of the NSSI group vs. 5.9% in the non-NSSI group (χ^2^ = 93.9, *p* < .001). Regarding sexual abuse, 28.9% in the NSSI group reported at least moderate to severe levels, while this was true for 6.7% in the non-NSSI group (χ^2^ = 130.5, p < .001). Concerning neglect, 33.7% in the NSSI group vs. 12.6% in the non-NSSI group reported moderate to severe emotional neglect (χ^2^ = 11.9, *p* < .05), while 36.2% in the NSSI group reported moderate to severe physical neglect vs. 22.0% in the non-NSSI group (χ^2^ = 111.3, p < .001). Further details on severity of neglect in association with NSSI can be seen in Fig. 1.

Participants with NSSI indicated significantly higher, yet on average not clinically relevant, scores of depression and anxiety symptoms. Furthermore, participants reporting NSSI were significantly younger and more likely to be female (for details see Table 2).

**Table 2:** Relevant characteristics of participants

	Total (*N* = 2498)	NSSI (*n* = 83)	No NSSI (*n* = 2415)	Chi^2^/T	Phi/ Cohen’s d
Age					
Mean (SD)	48.4 (18.2)	34.9 (14.4)	48.9 (18.2)	−6.93**	0.77
Range	14–94	15–79	14–94		
Gender				10.83*	0.07
Female (%)	1333 (53.4)	59 (71.1)	1274 (52.8)		
Male (%)	1165 (46.6)	24 (28.9)	1141 (47.2)		
Depression Score	0.69 (1.16)	2.26 (1.68)	0.64 (1.10)	12.71**	1.44
Anxiety Score	0.69 (1,14)	2.33 (1.78)	0.64 (1.07)	13.60**	1.54

**p < .001, *p < .05

### Path analytic model of child maltreatment related to NSSI, mediated by depression and anxiety scores

It was further hypothesized that (3) especially emotional abuse and neglect would directly affect NSSI, and (4) that physical abuse and –neglect, as well as sexual abuse, would be mediated by depression and anxiety symptoms.

**Fig. 2:**
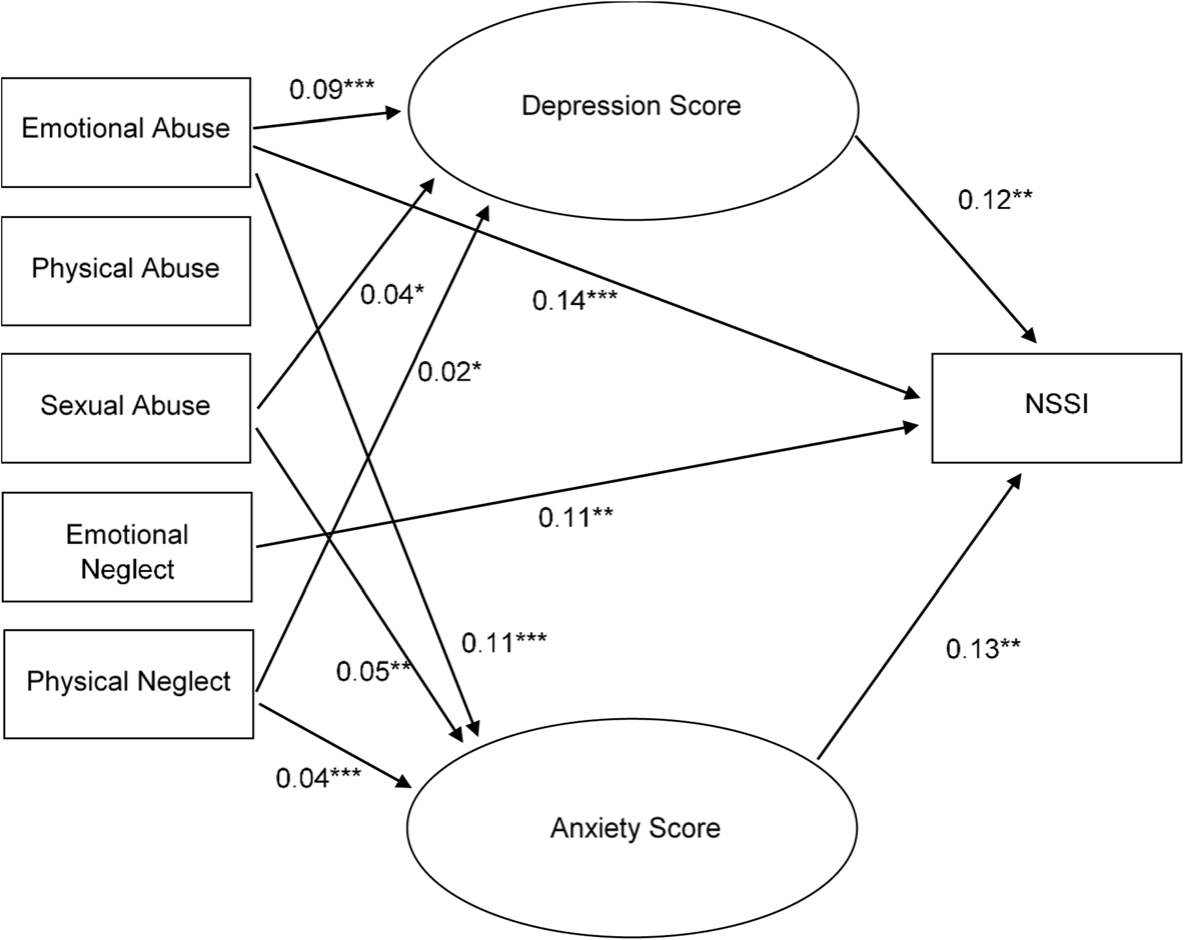
Path analytic model of child maltreatment related to NSSI with depression and anxiety scores as mediating factors. *Note*: For reasons of comprehensibleness, only significant associations are displayed in the graph. *p < .05, **p < .01, ***p < .001

The path analytic modelshowed good model fit (weighted root mean square residual (WRMS = .001, df = 59). Emotional abuse (estimated direct effect = 0.14, *p* < .001) and emotional neglect (estimated direct effect = 0.11, *p* = .001) were the only types of child maltreatment that showed significant direct effects on NSSI, while physical abuse (estimated direct effect = .05, *p* = .15), sexual abuse (estimated direct effect = .05, *p* = .08), and physical neglect (estimated direct effect = − 0.02, *p* = .75) did not. The effect of emotional neglect was not significantly mediated by depression or anxiety scores. The effect of emotional abuse was partially mediated by depression scores (specific indirect effect:=0.03, *p* = .02) and anxiety scores (specific indirect effect = 0.04, *p* = .01). Effects of sexual abuse and physical neglect were fully mediated by depression and anxiety scores, as direct effects of both variables on NSSI were non-significant, but indirect effects were(total indirect effect = 0.03, *p* = .009 for sexual abuse and total indirect effect: =0.02, *p* = .007 for physical neglect). Physical neglect was not significantly related to NSSI, depression or anxiety scores (for details see Fig. 2).

## Discussion

As former studies have reported conflicting results regarding the relationship between different types of abuse and neglect and NSSI, the aim of the current study was to investigate these associations as well as mediating effects of depression and anxiety on NSSI in the general population. In this representative sample of the German population, around 3% of all participants reported lifetime engagement in NSSI, and around 30% reported having experienced at least one form of child maltreatment. Among those participants with a lifetime history of NSSI, around 65% reported at least one type of maltreatment. Around 50% reported multiple types of maltreatment, while this was only true for around 12% of the general population. Overall, participants with a history of NSSI showed higher levels of severity of child maltreatment for all maltreatment subtypes (emotional, physical, and sexual abuse, emotional and physical neglect). However, a path analytic model showed only emotional neglect and abuse to be directly associated with NSSI, while the effect of sexual abuse and physical neglect were fully mediated by depression and anxiety symptoms.

Findings of this study are in line with previous findings reporting long-term negative mental health outcomes of child maltreatment [1]. Except physical abuse, all types of child maltreatment were significantly associated with higher depression and anxiety scores, as well as (sometimes indirectly) lifetime engagement in NSSI. This is the first study to report such long-term negative outcomes in a representative sample of the general population.

The association of child maltreatment and NSSI is especially obvious when considering that around two thirds of participants with NSSI reported child maltreatment, and 50% reported more than one type of maltreatment (of at least moderate to severe level). This could also point towards a dose-impact effect, meaning that the experience of several types of maltreatment might increase the likelihood of adverse long-term effects. A previous study also assessing child maltreatment using the CTQ [15], examined the relationship of child maltreatment with NSSI in the past year in a sample of 86 adolescents. Interestingly, even though the age difference between this sample and our sample was quite large, and NSSI within the past year vs. lifetime NSSI were assessed, results of both studies are rather similar. Results of the current study therefore add to the literature by showing that child maltreatment does not only have a rather short-term effect on NSSI during adolescence, but may have an effect on NSSI later on in life.

The types of maltreatment being directly associated with NSSI were a history of emotional abuse and neglect (72 and 65% of participants with NSSI reported at least mild-moderate emotional abuse and neglect, respectively, and 42 and 32% at least moderate-severe levels). This direct impact was still significant in the presence of depression or anxiety symptoms, while the effect of emotional abuse was partially mediated by depression and anxiety scores. Results concerning emotional abuse are in line with previous studies [10, 15, 35] and support theoretical approaches like the Developmental Psychopathology Network [21] or the Biopsychosocial Model by Marsha Linehan [36], both linking adverse childhood experiences with primary caregivers with the development of dysfunctional emotion regulation and thus developing dysfunctional coping skills like NSSI.

With regard to sexual abuse and physical neglect, the current study found support for those factors to play a role in the etiology of NSSI. However, these effects were fully mediated by the presence of depressive and anxiety symptoms. These results add on to findings (for review see [11, 17]) presenting sexual abuse as rather having an indirect than a direct effect on the development of NSSI. In previous studies, this effect was also mediated by factors like dissociation, self-esteem, or alexithymia, which were not assessed in the current study [12–15]. The fact that NSSI is most often engaged in as a dysfunctional emotion regulation strategy might explain why emotional abuse and –neglect were directly associated with NSSI, as they may more directly effect a persons’ ability to regulate emotions. On the other hand, physical types of abuse (like sexual or physical abuse) might only indirectly effect emotion regulation, for example in a person consequently developing symptoms of an affective disorder. This may have been one reason for effects of physical and emotional abuse being fully mediated by anxiety and depressive symptoms in the current study. However, assessment of other mediating factors, known to be linked to child maltreatment will be necessary before drawing further conclusions.

### Limitations

NSSI was only assessed by one question, which may have had an impact on the reliability of the assessment of NSSI. However, prevalence rates of NSSI in this study are in line with results of a previous, separate representative sample of the German population, reporting prevalence of lifetime NSSI of also around 3% [9]. While in the previous sample, NSSI was assessed much more rigorously by using the whole Self-Injurious-Thoughts-and-Behaviors Interview and assessing methods and functions of NSSI, the single assessment question in this study seemed to have been quite reliable as it yielded comparable rates. Furthermore, as this was a cross-sectional design assessing data in retrospect and in self-report, the possibility of memory bias regarding NSSI and child maltreatment has to be kept in mind when interpreting results. Regarding differential effect of types of maltreatment, one should be aware of the rather high rate of co-occurrence of different types of maltreatment. However, by entering all types of maltreatment into a path analytic model, this inter-correlation was accounted for. Furthermore, no corrections were made to protect against violation of assumptions in statistical tests due to the discrepant cell sizes between the two levels of the dichotomous outcome variable for NSSI.

## Conclusions

Child maltreatment, and especially emotional abuse and neglect, seem to play a significant role in the etiology of NSSI, above and beyond depressive and anxiety symptoms. Results of this study also have implications for preventative and therapeutic interventions. As particularly emotional abuse and neglect are oftentimes not detected (as they do not lead to physical indications like bruises and does not lead to a diagnosis of Posttraumatic Stress Disorder, as criterion A is not met), professionals working with children should be aware of its long-term consequences. This implies both, offering parenting training and supervision as preventative measures, and assessing and addressing emotional abuse and neglect in the treatment of NSSI. As impairment in emotion regulation seems to be a significant mediator of the effect between child maltreatment and NSSI, but is a complex concept to explore [22], it should be a focus in future studies. Future studies will also need to focus on outcomes of different types of child abuse and neglect on NSSI in a longitudinal design.

## Abbreviations

CTQ: Child Trauma Questionnaire
NSSI: Non-suicidal self-injury
